# A Comparative Study of the Anticancer Activity and PARP-1 Inhibiting Effect of Benzofuran–Pyrazole Scaffold and Its Nano-Sized Particles in Human Breast Cancer Cells

**DOI:** 10.3390/molecules24132413

**Published:** 2019-06-29

**Authors:** Manal M. Anwar, Somaia S. Abd El-Karim, Ahlam H. Mahmoud, Abd El-Galil E. Amr, Mohamed A. Al-Omar

**Affiliations:** 1 Department of Therapeutic Chemistry, National Research Centre, Dokki, Cairo 12622, Egypt; 2 Pharmaceutical Chemistry Department, Drug Exploration & Development Chair (DEDC), College of Pharmacy, King Saud University, Riyadh 11451, Saudi Arabia; 3 Applied Organic Chemistry Department, National Research Center, Cairo, Dokki 12622, Egypt

**Keywords:** breast cancer, benzofuran–pyrazole, nanoparticles, cytotoxic activity, apoptosis, PARP-1 inhibition

## Abstract

Breast cancer is considered the most common and deadly cancer among women worldwide. Nanomedicine has become extremely attractive in the field of cancer treatment. Due to the high surface to volume ratio and other unique properties, nanomaterials can be specifically targeted to certain cells and tissues to interact with the living systems. The strategic planning of this study is based on using the nanoprecipitation method to prepare nanoparticles **BZP-NPs** (3.8–5.7 nm) of the previously prepared benzofuran–pyrazole compound (**IV**) **BZP** which showed promising cytotoxic activity. The capacity of **BZP** and **BZP-NPs** to suppress the growth of human breast tumor MCF-7 and MDA-MB-231 cells was evaluated using MTT assay. The IC_50_ doses of **BZP** and **BZP-NPs** targeting normal breast cells MCF-12A exceeded those targeting the cancer cells by >1000-fold, demonstrating their reasonable safety profiles in normal cells. Furthermore, cell cycle analysis, apoptosis induction detection, assessment of p53, Bcl-2, caspase-3, and PARP-1 levels of **BZP** and its nano-sized-**BZP-NPs** particles were also evaluated. Although the obtained results were in the favor of compound **IV** in its normal-sized particles, **BZP-NPs** appeared as a hit compound which showed improved cytotoxicity against the tested human breast cancer cells associated with the induction of pre-G1 apoptosis as well as cell cycle arrest at G2/M phase. The increase in caspase-3 level, upregulation of p53, and downregulation of Bcl-2 protein expression levels confirmed apoptosis. Furthermore, ELISA results exhibited that **BZP-NPs** produced a more favorable impact as a PARP-1 enzyme inhibitor than the parent **BZP**.

## 1. Introduction

Breast cancer is the most common malignancy in women, accounting for about 18% of female cancers and over half a million new cases are diagnosed worldwide each year. Its incidence increases with age and is currently rising. Although earlier diagnosis has improved the survival rates, saving lives and elevating treatment rates, metastatic breast cancer is still considered as the major factor of breast cancer-related mortality [1,2,3,4]. Despite the continuous development in the treatment of cancer disease, the strategy for cancer management has remained essentially unchanged: surgical resection of the malignant tumor followed by either chemotherapeutic administration, radiotherapy, or a combination of the two. In fact, both of these therapies cause unselective and undesirable damage to the healthy tissues. In addition, a number of factors can lead to treatment failure, such as the remaining of some residual cells after the surgery, resistance to chemotherapies, physiological obstacles against the treatments, such as the blood–brain barrier and cellular barriers which hamper the access to drug targets, debilitating systemic toxicities, and poor pharmacokinetics of the chemotherapeutic [5,6,7].

Nanomedicine is defined as the use of nanotechnology for different medical purposes. Nanotechnology deals with research of materials of dimensions ranges between 1 to 100 nm (National Nanotechnology Initiative). This active protocol is applied in various science applications including cancer chemotherapeutics [8]. Deep studies have shed light on the combination of nanotechnology with cancer biology advances to gain novel techniques for cancer care. The concept that the strategy of nanomedicines is based on improving the therapeutic index of anticancer drugs by optimizing their pharmacokinetics and tissue distribution to facilitate and fasten delivery to the site of action is well known and has been investigated clinically [9,10,11]. Smaller (sub-100 nm) nanomedicine systems and lower molecular weight macromolecules have been shown to extravasate to a greater extent and/or penetrate farther from the vasculature than larger systems. This size effect has also been associated with improved efficacy [11,12]. Because of their sizes, the chemical properties and biodistribution of the nanomaterials are different from bulky materials. Thus, in recent years, nanomedicine has been considered as one of the most promising and important tools to defeat the problems obtained due to the administration of the traditional antitumor drugs. In addition, nanoparticle formulations can reduce or prevent systemic toxicities by specific delivery of the drugs to the cancer cells via size-mediated passive targeting and physiologically mediated active targeting. They can overcome drug resistance by the delivery of complimentary treatments and they can improve early detection of the disease using targeted delivery of molecular imaging agents to tumors in order to improve the diagnostic imaging of the tumor tissues, thus beginning the treatment before the onset of metastasis [13,14].

**Figure 1:**
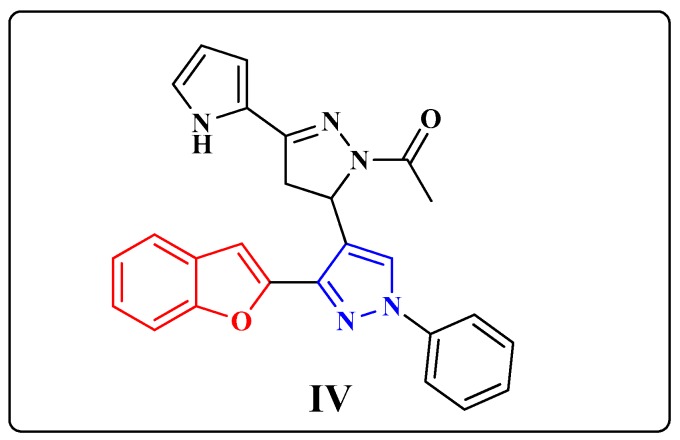
The chemical structure of the benzofuran-pyrazole compound **IV** (**BZP**).

Inspired by the literature studies mentioned above and in continuation with our previous efforts in developing new effective agents of significant anticancer activity [15,16,17], this study deals with generating the previously prepared 1-(5-(3-(benzofuran-2-yl)-1-phenyl-1*H*-pyrazol-4-yl)-4,5- dihydro-3-(1*H*-pyrrol-2-yl)pyrazol-1-yl)ethanone (**IV**) **BZP** in nanoparticles **BZP-NPs** of sizes 3.8–5.7 nm (Figure 1).

In our previous research, compound **IV** (**BZP**) was subjected, among other eight different benzofuran-pyrazole derivatives, to NCI for *in vitro* anticancer evaluation targeting full 60 human cancer cell lines using a single high dose concentration (10^−5^ M) under the drug discovery program of the NCI [15]. The derivatives were chosen depending upon the degree of structural variations and computer modeling techniques in NCI. Fortunately, compound **IV** (**BZP)** exhibited promising cytotoxic potency against various cancer cell lines, so it was further evaluated by NCI team at five different minimal concentrations (0.01, 0.1, 1, 10, and 100 µM). It displayed cell growth inhibition of different breast cancer lines in the range of 45.95–55.44%. These data motivated the authors to convert compound **IV** (**BZP**) to nano-sized **BZP-NPs** to study the influence of the nanorange and whether nano-sized particles enhance the cytotoxic potency of the benzofuran compound.

The anticancer activity of **BZP** compound **IV** was assessed in comparison with its nano-sized **BZP-NPs** against MCF-7 and MDA-MB-231cancer cell lines. Various cellular mechanisms of action were also studied, such as apoptosis, cell cycle analysis, detection of caspase-3, p53, and Bcl-2 intensities, in addition to the efficiency of PARP-1 enzyme inhibition in the two types of the tested breast cancer cell lines

## 2. Results and Discussion

### 2.1. Chemistry

**Scheme 1:**
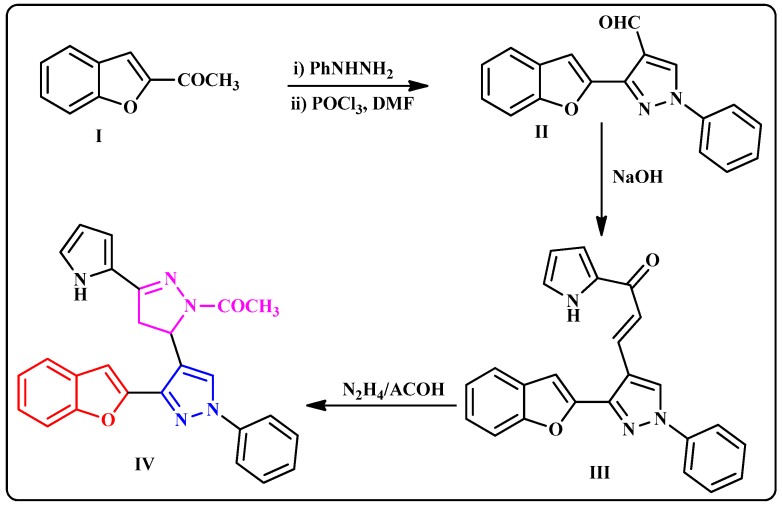
Synthetic route of the benzofuran–pyrazole derivative (**IV**).

The preparation approach of the benzofuran–pyrazole derivative **IV** was outlined in Scheme 1 according to the reported method [15]. Using the Vilsmeier–Haach reaction, the key starting 1-(benzofuran-2-yl)ethanone (**I**) was converted to the intermediate pyrazole-4-carbaldehyde (**II**). The chalcone analogue **III** was obtained in a good yield by Claisen–Schmidt condensation of **II** with 2-acetylpyrrole in ethanolic sodium hydroxide solution. Cyclocondensation of **III** with hydrazine hydrate in acetic acid yielded the target compound **IV** in 85% yield (Scheme 1).

**Figure 2:**
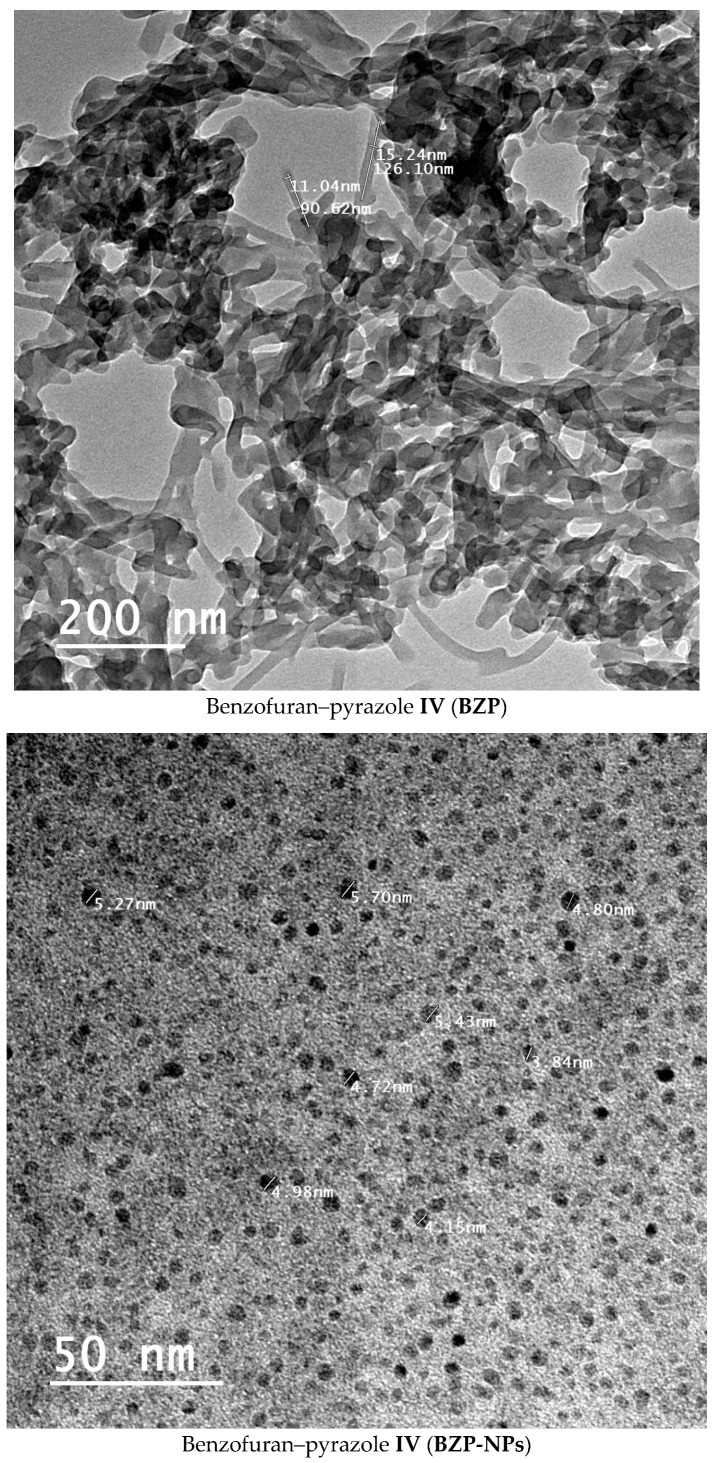
Electron micrograph of the **BZP** and **BZP-NPs**. The bar marker represents 50 nm.

The nano-sized benzofuran–pyrazole **BZP-NPs** of different sizes (3.8–5.7 nm) were synthesized using the nanoprecipitation method [18]. The sizes and morphology of the nanobenzofuran–pyrazole hybrid **BZP-NPs** were examined by dynamic light scattering (DLS) and transmission electron microscopy (TEM). The results showed that nanoparticles were spherical in shape and their average size was 3.8–5.7 nm (Figure 2). The stability of the **BZP-NPs** was further investigated by X-ray diffraction (XRD) using a Pananalylical Empyrean X-ray Diffractometer and thermal analysis using a SDT Q600 V20.9 Build 20 thermal gravimetric instrument (Figures S1 and S2, Supplementary material).

**Figure 3:**
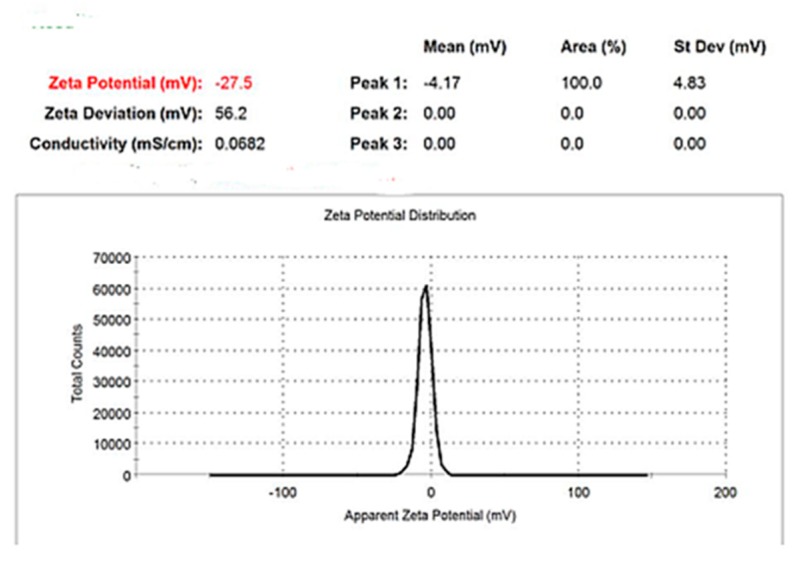
Zeta potential distribution of **BZP-NPs**.

Surface charge and stability of the nanoparticles were analyzed using the Malvern Zetasizer nano Zs instrument (MAL1074157) and the zeta potential was −27.3 mV with a polydispersity index (PDI) of 0.77 (Figure 3).

### 2.2. Biological Analysis

#### 2.2.1. In Vitro Anticancer Activity

The sensitivity of two human breast cancer cell lines, MCF-7 and MDA-MB-231, was evaluated against the benzofuran–pyrazole compound **BZP** and the target nano-sized benzofuran–pyrazole nanoparticles **BZP-NPs** using MTT assay. Doxorubicin served as a standard drug [17]. The resultant data were expressed as IC_50_ (nM) values which are the average of at least three independent experiments and are tabulated in Table 1. The obtained results showed that compound **IV** (**BZP**) produced significant cytotoxic activity against both breast cancer cell lines, with about 85- and 62-fold, respectively, greater potency than that of doxorubicin. Dramatic increase in the activity was observed by about 620 and 1000-fold for targeting both types of cancer cells by **BZP-NPs** compared to the reference drug doxorubicin. It could be detected that MDA-MB-231 cancer cells represented significant sensitivity against the nano-sized particles **BZP-NPs** more than their sensitivity against **BZP**.

Despite the obvious benefits of chemotherapeutic drugs, there are several treatment-related damages to the normal cells that should be considered before finalizing the cancer treatment strategy. This study investigated the impact of compound **IV** (**BZP**) and **BZP-NPs** on normal breast cells (MRC-12A) using MTT assay [16]. Interestingly, a significant increase in the IC_50_ doses of **IV BZP** and **BZP-NPs** against the normal breast cells was detected when compared to their IC_50_ doses against both cancer cell lines (>1000-fold) (Table 1). This result confirmed the significant safety profile of the benzofuran-pyrazole compound **IV** either in the normal size particles or in its nano-sized particles **BZP-NPs**.

#### 2.2.2. Cell Cycle Analysis

**Figure 4:**
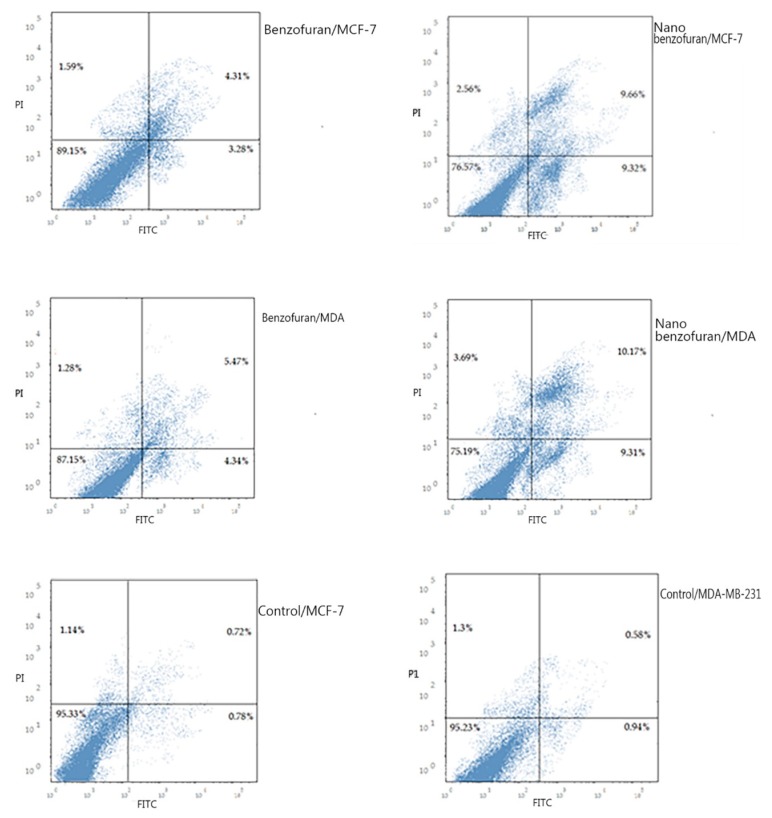
Representative dot plots of MCF-7 and MDA-MB-231 cells treated with compound **IV** (**BZP**) and **BZP-NPs** at their IC_50_ (µM) for 24 h, analyzed by flow cytometry after double staining of the cells with annexin-V FITC and PI.

**Figure 5:**
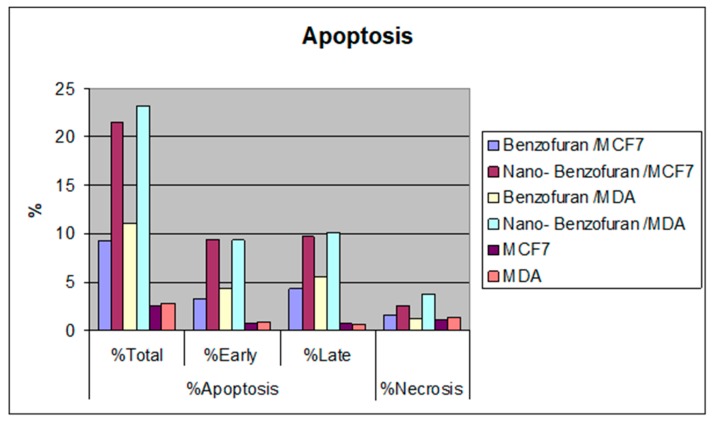
Percentage of compound **IV** (**BZP**) and **BZP-NPs** in MCF-7 and MDA-MB-231 cancer cells.

**Figure 6:**
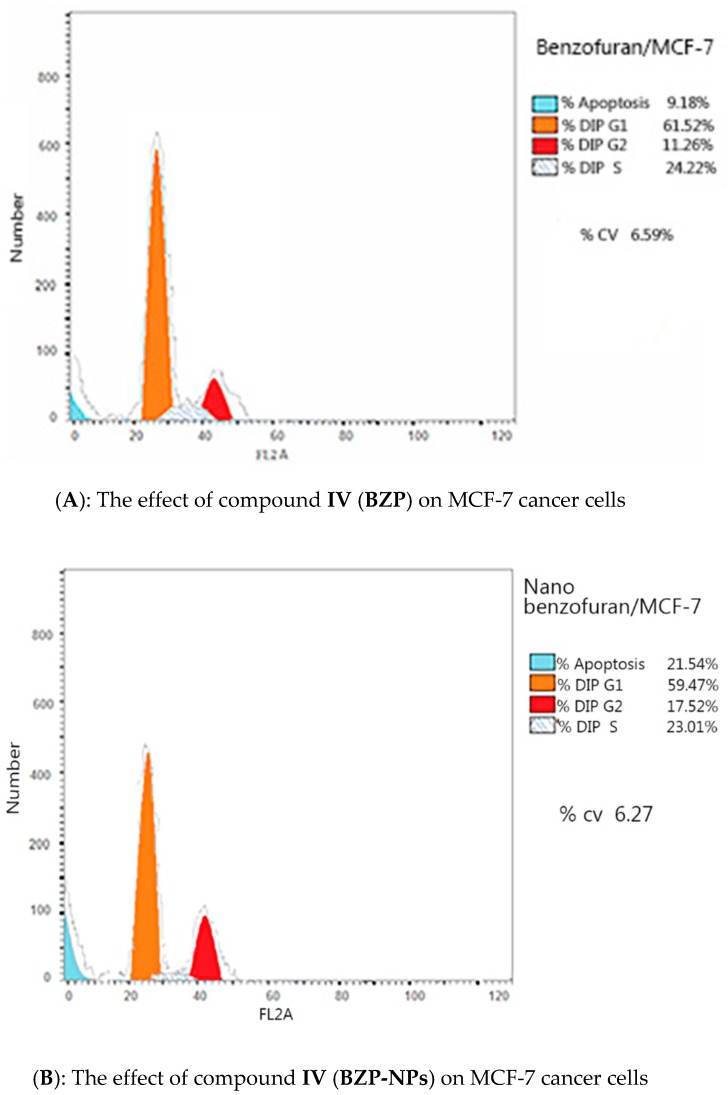

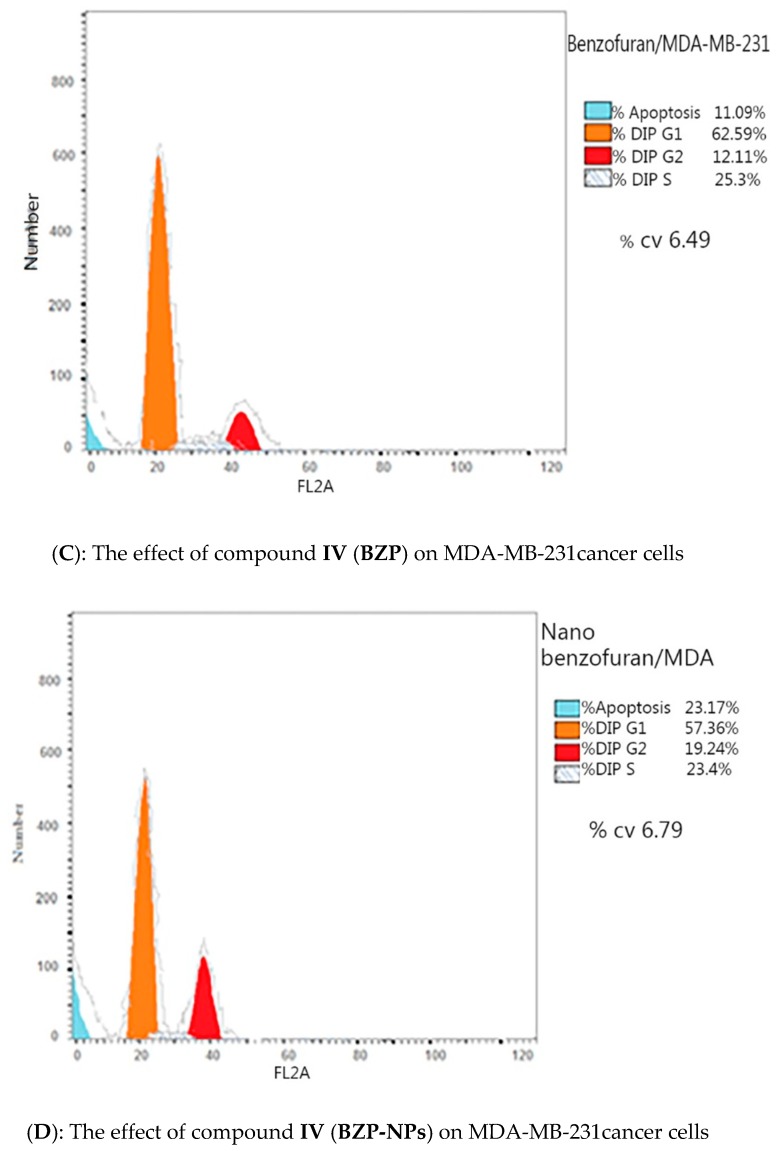

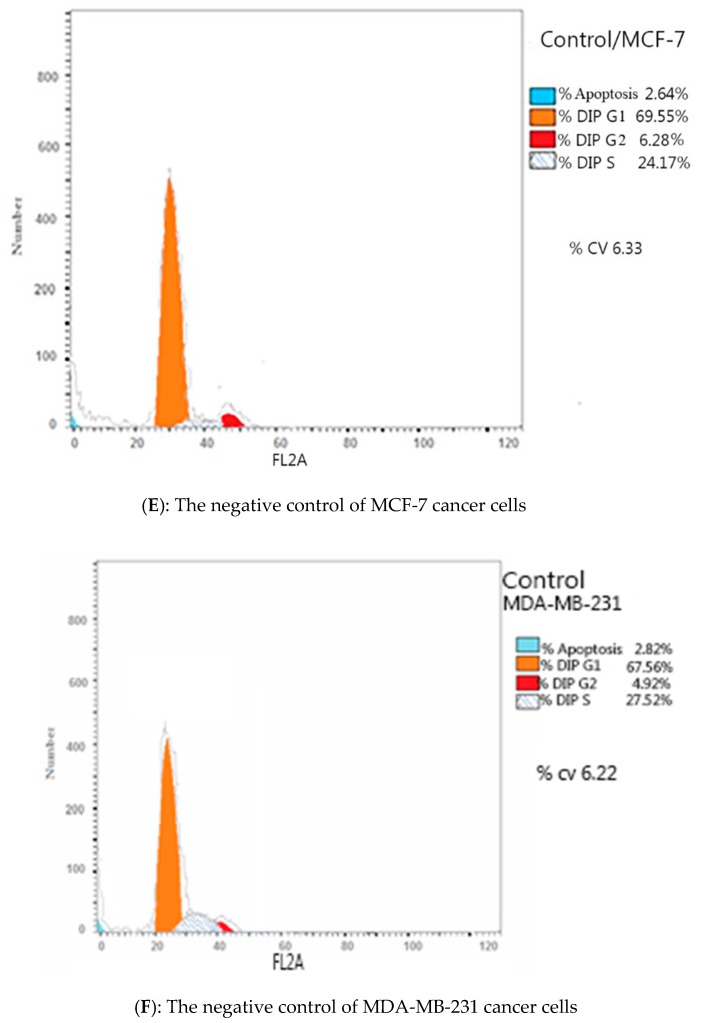
Flow cytometric analysis of compound **IV** (**BZP**) and **BZP-NPs** on MCF-7 and MDA-MB-231 cells. The orange color represents G1 phase percentage and red color represents G2/M phase percentage.

**Figure 7:**
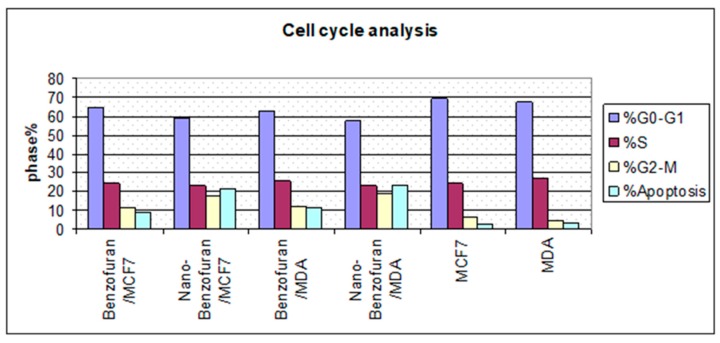
Cycle analysis of compound **IV** (**BZP**) and **BZP-NPs** in MCF-7 and MDA-MB-231 cancer cells.

Due to the antiproliferative efficacy of compound **IV** (**BZP**) and **BZP-NPs**, it was of interest to study its modes of action in both tested types of cancer cells, including cell cycle progression and apoptosis induction. Cell death occurs via different pathways, including apoptosis or type I cell-death, and autophagy or type II cell-death, which are both forms of programmed cell death, whereas necrosis is a nonphysiological process resulting from an infection or injury [19,20]. Apoptosis rate in MCF-7 and MDA-MB-231 cells was detected by flow cytometry, using propidium iodide (PI) and an annexin-V–FITC double staining assay [21]. After incubation of both types of the tested cancer cells for 24 h with BZP and BZP-NPs at their IC_50_ concentrations of 7 nM and 1 nM for MCF-7 cells and at 10 nM and 0.6 nM for MDA-MB-231 cells, they were labeled with the two dyes. The corresponding red (PI) and green (FITC) fluorescence was detected using flow cytometry and the results were compared to DMSO-treated cells which served as a negative control. Marked alterations in cell cycle phases have occurred. There was a great enhancement in the percentage of apoptotic cells at the pre-G phase. The tested particles (**BZP**) and (**BZP-NPs**) induced total apoptotic cells (annexin V^+^/PI^−^ and annexin V^+^/PI^+^) of percentages 9.18%, 21.54%, respectively, in MCF-7 cells vs. 2.64% in the control MCF-7 cells. They also induced total apoptotic percentages of 11.09% and 23.17%, respectively, in MDA-MB-231 cells vs. 2.82% in the control MDA-MB-231cells. It is observable that the **BZP-NPs** produced a more favorable impact about two-fold more potent than **BZP** particles against both types of the tested cancer cells, especially MDA-MB-231 cells, which was consistent with the cytotoxicity assay results (Figure 4 and Figure 5). Also, exposure of MCF-7 and MDA-MB-231 cells to **BZP** and **BZP-NPs** led to an interference with the cell cycle distribution, inducing a pronounced elevation in the percentage of cells at the G2/M phase, reaching 11.26% and 17.52%, respectively, in MCF-7 vs. 6.28% in the control cells and to 12.11% and 19.24% in MDA-MB-231 cells, respectively, vs. 4.92% in the negative control (Table 2). Accordingly, it can be concluded that the compound **IV** (**BZP)** inhibits the cancer cells’ proliferation with a synchronous significant arrest at the G2/M phase. The inhibition potency was signified by the **BZP-NPs** (Figure 6 and Figure 7). Accumulation of cells at G2/M phase is a remarkable hallmark of the apoptotic role of **BZP** and **BZP-NPs** in both tested cancer cell lines.

#### 2.2.3. Effect Compound **IV** (**BZP**) and **BZP-NPs** on the Levels of Caspase-3/p53/Bax/Bcl-2

Caspases are cysteine protease enzymes in humans. Their presence is critical in starting the phase of programmed cell death (apoptosis). Some caspases initiate the intracellular cascade, whereas others (effector caspases) produce their activities downstream by controlling the cellular break via splitting of the structural proteins [19,20,21]. Caspase-3 displays an important role in the apoptotic process which includes cell shrinkage, chromatin condensation, and DNA fragmentation [22,23]. Enzyme Linked Immuno-Sorbent Assay (ELISA) was used to analyze the apoptotic events of both types of the tested breast cancer cells [16]. Table 3 shows that 24 h treatment of MCF-7 with compound **IV** (**BZP**) and **BZP-NPs** at concentrations of 7 nM and 1 nM and MDA-MB-231 cells at concentrations of 10 nM and 0.6 nM led to significant overexpression of caspase-3 compared to the doxorubicin-treated cells. **BZP** elevated the level of caspase-3 by six and five-fold compared to the untreated cells. A detectable enhancement in the level of caspase-3 occurred upon treatment of the tested cells with **BZP-NPs** by 14 and 17-fold compared to the untreated MCF-7 and MDA-MB-231 cells (Table 3).

The tumor suppressor protein p53 serves as a transcription factor. It induces the expression of several downstream targets which are very important in regulation of the cell cycle, apoptosis, and DNA repair, among other mechanisms [24,25]. Cellular stress leads to the activation of the p53 pathway, compromising tumor development and preventing the proliferation of the damaged cells of oncogenic potential. p53 is considered as one of the most relevant tumor suppressor genes [26]. In addition, the B-cell lymphoma protein 2 (Bcl-2) plays a key role in tumor progression via inhibition of the intrinsic apoptotic pathway triggered by mitochondrial dysfunction. Cancer cells can resist apoptosis by modulating the expression of Bcl-2 family proteins which in turn regulate the mitochondrial apoptotic pathway via production Bcl-2 or downregulating pro-apoptotic proteins, such as Bax [26]. Accordingly, in this study, the impact **of IV** (**BZP**) and **BZP-NPs** was assessed on the intrinsic apoptotic pathway via measuring the levels of p53, Bax, and Bcl-2 after treatment of MCF-7 and MDA-MB-231 cells with **BZP** and **BZP-NPs** at their IC_50_ concentrations for 24 h. In comparison to the untreated control, p53 level increased seven-fold in both types of the tested cancer cells upon **BZP** treatment, while the level was doubled to 14-fold by the **BZP-NPs**. On the other hand, **BZP** and **BZP-NPs** elevated the level of the proapoptotic protein Bax by 5.7–13.1-fold with concurrent reduction in the expression levels of the antiapoptotic protein Bcl-2 by four to seven-fold in both tested cancer cell lines in comparison with the untreated control (Table 3).

#### 2.2.4. PARP-1 Cleavage Assay

Breast cancer is among the targets of a new class of drugs known as Poly (ADP-ribose) polymerase-1 (PARP-1) inhibitors. PARP-1 is a nuclear enzyme plays a role in the repair of single-stranded DNA (ss DNA) breaks [26]. The rationale for the therapeutic benefit for the pharmacological inhibition of PARP-1 in breast cancer comes from the following points: (a) sensitization of tumor cells to anticancer therapies such as radiation and cytotoxic agents [27]; (b) certain polymorphisms in PARP-1 can lead to breast cancer and negatively affect the efficacy of the hormone therapies; and (c) breast tumors with deficiencies in DNA-repair genes such as BRCA-1 or BRCA-2 represent acute sensitivity in response to the inhibition of PARP-1. Interestingly, clinical evidence investigates that the usage of PARP-1-inhibiting candidates are not limited to BRCA-1 or BRCA-2 mutated cancers, but they also target non-BRCA mutated breast and ovarian cancers and produce a valuable impact in combination therapy [28,29]. Thus, it was of interest to study the inhibitory effects of **BZP** and **BZP-NPs** against PARP-1 enzyme in MCF-7 and MDA-MB-231 cancer cells using staurosporine as a standard drug. The resulting data were expressed as IC_50_ (nM) values and are summarized in (Table 4). It should be noted that **BZP** produced a slightly weaker inhibitory effect against PARP-than the reference drug, with an IC_50_ of 40 nM vs. 10 nM for staurosporine in MCF-7 cells. A dramatic decrease, about 13-fold, in the sensitivity was noticed in the case of MDA-MB-231 cancer cells against **BZP** which produced an IC_50_ of 60 nM vs. 8 nM for staurosporine. On the other hand, an interesting increase in the inhibitory potency was observed for the **BZP-NPs**, exhibiting an IC_50_ value of 10 nM, which is equal to that obtained by the standard drug in MCF-7 cells. The suppression activity was intensified against PARP-1 in MDA-MB-231 cells, producing an IC_50_ of 6 nM vs. 8 nM for staurosporine.

## 3. Experimental

### 3.1. Synthesis of 1-(5-(3-(Benzofuran-2-yl)-1-phenyl-1H-pyrazol-4-yl)-4,5-dihydro-3-(1H-pyrrol-2-yl) pyrazol-1-yl)ethanone (***IV***). 

Compound **IV** was synthesized according to the previously reported procedure [15].

### 3.2. Preparation of Nanobenzofuran–Pyrazole ***BZP-NPs***

The nanoparticles were prepared by the nanoprecipitation method [18]. The sizes and morphology of the nanoparticles **BZP-NPs** were examined by transmission electron microscopy (TEM) (H-7600; Hitachi Ltd., Tokyo, Japan). The results exhibited that the nanoparticles were spherical in shape and their average size was 3.8–5.7 nm (Figure 2). 

### 3.3. Physicochemical Characterization of the Nanobenzofuran–Pyrazole Compound ***BZP-NPs***

#### 3.3.1. Particle Size and Zeta Potential Using Photon Correlation Spectroscopy 

Particle size was measured by dynamic light scattering (DLS) using a Zetasizer NANO-ZS (Ver. 7.04, Serial Number: MAL 1074157, Malvern Instruments Ltd., London, United Kingdom) at a wavelength of 633 nm with a 4.0 mW light source for collecting data at a fixed scattering angle of 173°. The electrophoretic mobility (zeta potential) measurements were made using a Zetasizer NANO-ZS (Ver. 7.04, Serial Number: MAL 1074157, Malvern Instruments Ltd., United Kingdom) at 25 °C. **BZP-NPs** morphology was determined using JEOL Transmission Electron Microscope (JEM-1230, Tokyo, Japan) with 500,000 × magnification power, 100 kV acceleration voltage, and 0.5 nm resolving power. 

#### 3.3.2. In Vitro Anticancer Activity

In vitro evaluation of the anticancer activity of the **BZP** compound and **BZP-NPs** targeting MCF-7 and MDA-MB-231 cells was performed using MTT assay according to a previously reported method [19]. Each experiment was performed at least three times. 

### 3.4. Cell Cycle Analysis and Apoptosis Detection

Cell cycle analysis and apoptosis detection was carried out by flow cytometry (Beckman Coulter, Brea, CA, USA) [19,20,21]. Apoptosis detection was performed using a FITC Annexin-V/PI commercial kit (Becton Dickenson, Franklin Lakes, NJ, USA) following the manufacturer’s protocol. 

### 3.5. Caspases-3 Assays

Caspase-3 activity was measured using a Caspase-3 (Active) (human) ELISA kit, Catalog # KHO1091 (96 tests) (Invitrogen Corporation, Carlsbad, CA, USA) according to the manufacturer’s instructions [16].

### 3.6. In Vitro Determination of p53, Bax, and Bcl-2 Levels

The levels of p53, Bax, and Bcl-2 markers were assessed using a BIORAD iScript TM One-Step RT-PCR kit with SYBR^®^ Green according to the manufacturer’s instructions [26,30]. 

### 3.7. In Vitro PARP-1 Assay

The procedure was done according to the supplied protocol of ab119690 Cleaved PARP Human ELISA (Enzyme-Linked Immunosorbent Assay) kit for the quantitative measurement of the 89 kDa fragment of Human PARP-1 in cell and tissue lysates [31].

## 4. Conclusions 

This study demonstrates that the conversion of the benzofuran-pyrazole compound **IV** (**BZP**) to nanoparticles **BZP-NPs** greatly intensified its cytotoxic activity against two breast cancer cell lines, MCF-7 and MDA-MB-231, with respective IC_50_ values of 7 nM, 10 nM vs. nanoparticle IC_50_ values of 1 nM, 0.6 nM. The IC_50_ value of DOX was 620 nM. Furthermore, the IC_50_ doses of BZP and BZP-NPs against normal breast cells were >1000-fold greater than those against cancer cells, suggesting acceptable safety profiles in normal cells. The resultant data of cell cycle and apoptosis determination revealed that the tested derivative in both forms induced G2/M phase arrest, accompanied by an increase in apoptosis in the tested cancer cells. Further modes of action of the target compound were also predicted in both types of breast cancer cells. The biological results revealed that **BZP** significantly increased p53, caspase-3, and Bax levels and decreased Bcl-2 levels, and their levels were intensified upon treating the tested cancer cells with (**BZP-NPs**). The PARP1 enzyme assay showed that the efficiency of PARP-1 inhibition by (**BZP**) was slightly less than that of staurosporine, while **BZP-NPs** inhibited the enzyme efficiently as staurosporine in MCF-7 or to a greater extent in case of MDA-MB-231 cancer cells. It has been detected that the nanoparticles were more effective as an anticancer agent against MDA-MB-231 cells than against MCF-7 cancer cells.

## Figures and Tables

**Table 1:** In vitro cytotoxic activity of compound **IV** (**BZP**) and **BZP-NPs**.

Compound Name	IC_50_ (nM)		
	MCF-7	MDA-MB-231	MCF-12A
Compound IV (**BZP**)	7 ± 1	10 ± 1	87600 ± 335
Compound IV (**BZP-NPs**)	1 ± 0.4	0.6 ± 0.1	21540 ± 66
Doxorubicin	620 ± 31	620 ± 31	

**Table 2:** Determination of cell cycle inhibition of MCF-7 and MDA-MB-231 cancer cells by **BZP** and **BZP-NPs**.

Compound Name	Conc. (nM)	%G0-G1	%S	%G2-M	%Pre-G1	Comment
**BZP**/MCF-7	7	64.52	24.22	11.26	9.18	PreG1apoptosis&Cell growth arrest@ G2/M
**BZP-NPs**/MCF-7	1	59.47	23.01	17.52	21.54	PreG1apoptosis&Cell growth arrest@ G2/M
**BZP**/MDA-MB-231	10	62.59	25.3	12.11	11.09	PreG1apoptosis&Cell growth arrest@ G2/M
**BZP-NPs**/MDA-MB-231	0.6	57.36	23.4	19.24	23.17	PreG1apoptosis&Cell growth arrest@ G2/M
MCF-7		69.55	24.17	6.28	2.64	
MDA-MB-231		67.56	27.52	4.92	2.82	

**Table 3:** Determination of caspase-3, p53, Bax, and Bcl-2 levels in the tested cancer cells.

Results (Fold Change)				
Compound Name	Caspase-3	p53	Bax	Bcl_-_2
**BZP**/MCF-7	6.383836	7.453852	5.745321	0.272695
**BZP-NPs**/MCF-7	14.56524	12.51432	9.149760	0.131011
**BZP**/MDA-MB-231	5.399087	7.792609	7.553853	0.181989
**BZP-NPs**/MDA-MB-231	17.915	14.60536	13.19230	0.134738
MCF-7	1	1	1	1
MDA-MB-231	1	1	1	1

**Table 4:** In vitro PARP-1 inhibitory assay of compound **IV (BZP)** and **BZP-NPs**.

Compound Name	IC_50_ (nM)	
	MCF-7	MDA-MB-231
**BZP**	40 ± 1	60 ±1
**BZP-NPs**	10 ± 4	6 ± 3
Staurosporine	10 ± 1	8 ± 1

## Supplementary Materials

The following are available online, Figure S1: Diffraction (XRD) of BZP-NPs, Figure S2: Analysis of **BZP-NPs**.
