# Supplementary material for: A Comparative Study of the Anticancer Activity and PARP-1 Inhibiting Effect of Benzofuran–Pyrazole Scaffold and Its Nano-Sized Particles in Human Breast Cancer Cells
Source: Molecules. 2019 Jun 29;24(13):2413. doi: 10.3390/molecules24132413 (PMC6650948; doi:10.3390/molecules24132413)
Supplement: Supplementary file 1 [file molecules-24-02413-s001.pdf]

3. Experimental

3.1. Preparation of the Nanobenzofuran–Pyrazole Compound (BZP-NPs)

The nanoparticles were prepared by the nanoprecipitation method. Four mg of the aqueous compound **BZP** was dissolved in 1 mL of distilled water and well stirred for 15 minutes. The pH of the solution was made acidic by adding 1 M HCl dropwise. With continuous stirring, 10 m L of isopropyl alcohol was added dropwise to the previous compound solution at room temperature. The solution was left while stirring for 2 h, then centrifuged at 13,000 rpm for 5 min, to obtain the pellet. The obtained pellet was washed thrice using distilled water and PBS was added to neutralize the pH. The dry weight of the pellet was noted to determine the yield of the nanoparticles prepared. The yield was calculated as the percentage dry weight of the nanoparticle pellet formed to the weight of **BZP** taken for the preparation. The pellet was then resuspended in distilled water and kept for lyophilization. The lyophilized powder was used for further characterization and cell studies. The sizes and morphology of the nanoparticles **BZP-NPs** were examined by transmission electron microscopy (TEM) (H-7600; Hitachi Ltd, Tokyo, Japan). The results showed that the nanoparticles were spherical in shape and their average size was 3.8–5.7 nm (Figure 2).

3.3. Physicochemical Characterization of the Nanobenzofuran–Pyrazole Compound **BZP-NPs**

3.3.1. Particle Size and Zeta Potential Using Photon Correlation Spectroscopy

Particles nano-size was measured by dynamic light scattering (DLS) using Zetasizer NANO-ZS (Ver. 7.04, Serial Number: MAL 1074157, Malvern Instruments Ltd., London United Kingdom) at the wavelength of 633 nm and a power of 4.0 mW as a light source collecting data at a fixed scattering angle of 173°. The electrophoretic mobility (zeta potential) measurements were made using Zetasizer NANO-ZS (Ver. 7.04, Serial Number: MAL 1074157, Malvern Instruments Ltd., United Kingdom) at 25 °C. BZP-NPs morphology was determined using JEOL Transmission Electron Microscope (JEM-1230, Tokyo, Japan, with 500.000 × magnifica-tion power, 100 KV acceleration voltages and 0.5 nm resolving power).

3.3.2. *In Vitro* Anticancer Activity

MTT assay was used to evaluate the in vitro cytotoxicity of the **BZP** compound and **BZP-NPs** against the breast cancer cell lines MCF-7 and MDA-MB-231 [19]. The MTT assay depends on the reduction of the soluble 3-(4,5-methyl-2-thiazolyl)-2,5-diphenyl-2H-tetrazolium bromide (MTT) into a blue purple formazan product, mainly by mitochondrial reductase activity inside living cells. The cells used in cytotoxicity assays were cultured in RPMI 1640 medium supplemented with 10% fetal calf serum. Cells suspended in the medium (2×10^4 cells/mL) were plated in 96-well culture plates and incubated at 37 °C in a 5% CO_2 incubator. After 12 h, the test sample (2 μL) was added to the cells (2×10^4) in 96-well plates and cultured at 37 °C for 3 days. The cultured cells were mixed with 20 μL of MTT solution and incubated for 4 h at 37 °C. The supernatant was carefully removed from each well and 100 μL of DMSO was added to each well to dissolve the formazan crystals which were formed by the cellular reduction of MTT. After mixing with a mechanical plate mixer, the absorbance of each well was measured by a microplate reader using a test wavelength of 570 nm. The results were expressed as the IC_{50} (μM), which induced a 50% inhibition of cell growth of the treated cells when compared to the growth of control cells. Each experiment was performed at least three times. There was a good reproducibility between the replicate wells with standard errors.

3.4. Cell Cycle Analysis and Apoptosis Detection

Cell cycle analysis and apoptosis investigation were carried out by flow cytometry [1–3]. MCF-7 and MDA-MB-231 cells were seeded into six well plates at a density of 2×10^5 per well and incubated

at 37 °C, 5% CO_2 for 24 h. After treatment with the tested compound for 24 h, cell pellets were collected and centrifuged (300× *g*, 5 min). For cell cycle analysis, cell pellets were fixed with 70% ethanol on ice for 15 min and collected again. The collected pellets were incubated with propidium iodide (PI) staining solution (50 mg/mL PI, 0.1 mg/mL RNaseA, 0.05% Triton X-100) at room temperature for 1 h and analyzed by a Gallios flow cytometer (Beckman Coulter, Brea, CA, USA). Apoptosis detection was performed by FITC Annexin- V/PI commercial kit (Becton Dickenson, Franklin Lakes, NJ, USA) following the manufacturer's protocol. The samples were analyzed by fluorescence-activated cell sorting (FACS) with a Gallios flow cytometer (Beckman Coulter, Brea, CA, USA) within 1 h after staining. Data were analyzed using Kaluza v 1.2 (Beckman Coulter).

3.5. Caspases-3 Assays

Cell line cells of MCF-7 and MDA-MB-231 were obtained from ATCC. RPMI 1640 medium supplemented with 10% fetal calf serum was used to allow cells to grow 37 °C, stimulated with the compound tested for caspases-3 and lysed with Cell Extraction Buffer. Standard Diluent Buffer was used to dilute the lysate over the range of the assay and measure human active caspase-3 content. Cells were plated at a density of 1.2–1.8 × 10,000 cells/well in a volume of 100 μL complete growth medium + 100 μL of the tested compound per well in a 96-well plate for 24/48 h before the enzyme assay [4].

3.6. In Vitro Measurement of p53 and Bcl-2 Protein Concentration

The levels of the tumor suppressor gene p53 and the antiapoptotic marker Bcl-2 were assessed using a p53 ELISA kit and a Bcl-2 Elisa kit, respectively. The procedure of the used kits was done according to the manufacturer's instructions. Briefly, cell lysates were prepared from control, MCF-7, and MDA-MB-231 cells (2.5×10^5/mL) treated with the IC_{50} concentration of the tested compound. Then, equal amounts of cell lysates were loaded then probed with specific antibodies. The samples were measured at λ 450 nm in ROBONEK P2000 ELISA reader. Analysis was confirmed with three different sets of extracts. All experiments were performed in triplicate [5,6].

3.7. In Vitro PARP-1 Assay

The procedure was done according to the supplied protocol of ab119690 Cleaved PARP Human ELISA (Enzyme-Linked Immunosorbent Assay) kit for the quantitative measurement of the 89 kDa fragment of Human PARP-1 in cell and tissue lysates, including lysates of adherent and suspension cells grown in 96-well microplate format and lysed directly in culture media.

By interpretation of data, the IC_{50} of the compound can be calculated in comparison to staurosporine as a standard reference [7].

Anchor Scan Parameters

Dataset Name:	1
File name:	C:\XRD Data\Services\ManalAnoar\1.xrdml
Comment:	Configuration=Flat Sample Stage, Owner=User-1, Creation date=2/9/2011 11:00:54 AM<br>Goniometer=Theta/Theta; Minimum step size 2Theta:0.0001; Minimum step size Omega:0.0001<br>Sample stage=Stage for flat samples/holders<br>Diffractometer system=EMPYREAN<br>Measurement program=Aisha, Owner=User-1, Creation date=6/20/2011 11:07:36 AM
Measurement Date / Time:	6/16/2019 2:12:33 PM

Operator:	TEST1
Raw Data Origin:	XRD measurement (*.XRDML)
Scan Axis:	Gonio
Start Position [°2Th.]:	4.0131
End Position [°2Th.]:	79.9591
Step Size [°2Th.]:	0.0260
Scan Step Time [s]:	18.8700
Scan Type:	Continuous
PSD Mode:	Scanning
PSD Length [°2Th.]:	3.35
Offset [°2Th.]:	0.0000
Divergence Slit Type:	Fixed
Divergence Slit Size [°]:	0.0573
Specimen Length [mm]:	10.00
Measurement Temperature [°C]:	25.00
Anode Material:	Cu
K-Alpha1 [Å]:	1.54060
K-Alpha2 [Å]:	1.54443
K-Beta [Å]:	1.39225
K-A2 / K-A1 Ratio:	0.50000
Generator Settings:	30 mA, 45 kV
Diffractometer Type:	0000000011089631
Diffractometer Number:	0
Goniometer Radius [mm]:	240.00
Dist. Focus-Diverg. Slit [mm]:	100.00
Incident Beam Monochromator:	No
Spinning:	No

Graphics

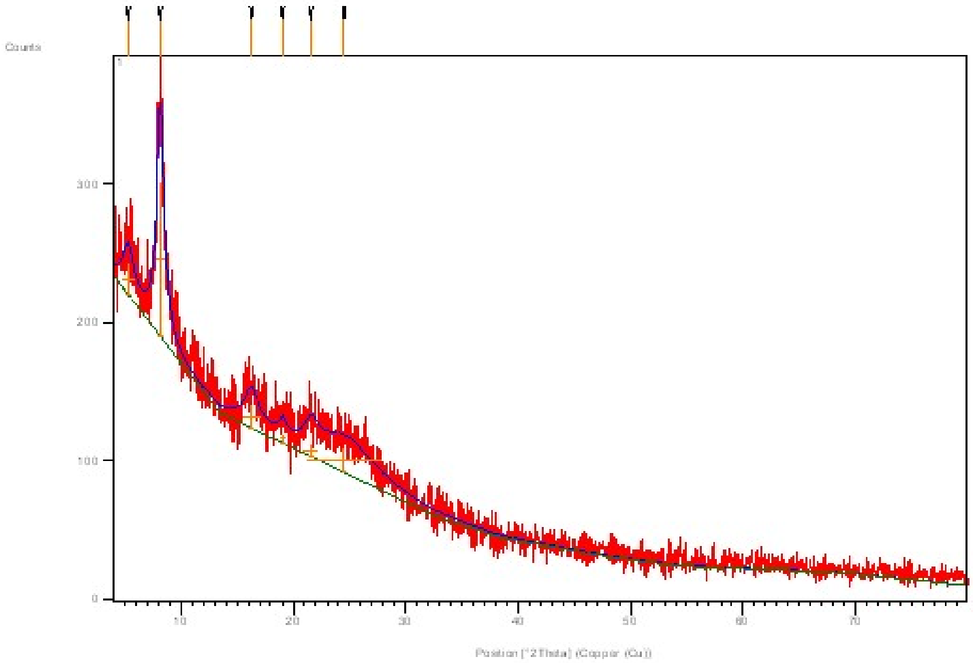

Figure S1. Diffraction (XRD) of **BZP-NPs**

Peak List

Pos.[°2Th.]	Height [cts]	FWHMLeft[°2Th.]	d-spacing [Å]	Rel. Int. [%]
5.33(7)	23(3)	1.2(3)	16.57826	20.26
8.18(1)	112(4)	0.81(5)	10.79464	100.00
16.27(8)	17(2)	1.5(3)	5.44368	15.28
19.0(1)	7(3)	0.7(2)	4.65792	6.10
21.6(1)	9(3)	1.3(4)	4.11624	8.49
24.5(4)	18(1)	7(1)	3.63058	16.10

Document History

Insert Measurement:
- File name = "1.xrdml"
- Modification time = "6/19/2019 2:17:33 PM"
- Modification editor = "TEST1"

Default properties:
- Measurement step axis = "None"
- Internal wavelengths used from anode material: Copper (Cu)
- Original K-Alpha1 wavelength = "1.54060"
- Used K-Alpha1 wavelength = "1.54060"

- Original K-Alpha2 wavelength = "1.54443"
- Used K-Alpha2 wavelength = "1.54443"
- Original K-Beta wavelength = "1.39225"
- Used K-Beta wavelength = "1.39225"
- Irradiated length = "10.00000"
- Spinner used = "No"
- Receiving slit size = "0.10000"
- Distance to sample = "Diffracted radius"
- Step axis value = "0.00000"
- Offset = "0.00000"
- Sample length = "10.00000"
- Modification time = "6/19/2019 2:17:33 PM"
- Modification editor = "TEST1"

Interpolate Step Size:
- Derived = "Yes"
- Step Size = "0.01"
- Modification time = "6/19/2019 2:17:33 PM"
- Modification editor = "PANalytical"

Determine Background:
- Correction method = "Manual"
- Use cubic spline interpolation = "No"
- Background base points = "4.01313028 233.999180111204,13.3991302799999 135,32.4311302799998 61,37.9171302799997 46,44.4431302799997 36,50.5531302799996 28,54.9991302799996 24,69.4551302799995 9.20427153799251,79.9591302799994 10.1014341635776"
- Modification time = "6/19/2019 2:17:50 PM"
- Modification editor = "TEST1"

Search Peaks:
- Minimum significance = "3"
- Minimum tip width = "0.05"
- Maximum tip width = "1"
- Peak base width = "2"
- Method = "Minimum 2nd derivative"
- Modification time = "3/23/2015 1:21:51 PM"
- Modification editor = "TEST1"

Insert Peak:
- Peak position [°2Th.] = "21.5975"
- Modification time = "6/19/2019 2:17:59 PM"
- Modification editor = "TEST1"

Insert Peak:
- Peak position [°2Th.] = "24.8647"
- Modification time = "6/19/2019 2:18:01 PM"
- Modification editor = "TEST1"

Insert Peak:
- Peak position [°2Th.] = "16.2512"
- Modification time = "6/19/2019 2:18:02 PM"
- Modification editor = "TEST1"

Insert Peak:
- Peak position [°2Th.] = "19.3699"
- Modification time = "6/19/2019 2:18:03 PM"
- Modification editor = "TEST1"

Insert Peak:
- Peak position [°2Th.] = "5.2616"
- Modification time = "6/19/2019 2:18:05 PM"
- Modification editor = "TEST1"

Edit Method:
- Old Value = "Polynomial"
- Modification time = "6/19/2019 2:18:20 PM"
- Modification editor = "TEST1"

Profile fitting:
- Angular range [°2Th.] = "4.013 - 79.959"
- Step No. 1
- Title = "Flat Background"
- Min. Shift/ESD = "0.1"
- Switch off after usage = "False"
- Step No. 2
- Title = "More background"
- Min. Shift/ESD = "0.1"
- Switch off after usage = "False"
- No. additional parameters = "3"
- Use 1/X background too = "True"
- Step No. 3
- Title = "Peak Position"
- Min. Shift/ESD = "0.1"
- Switch off after usage = "False"
- Step No. 4
- Title = "Peak Height"

- Min. Shift/ESD = "0.1"
- Switch off after usage = "False"
- Step No. 5
- Title = "Peak FWHM"
- Min. Shift/ESD = "0.1"
- Switch off after usage = "False"
- Step No. 6
- Title = "Peak Shape"
- Min. Shift/ESD = "0.1"
- Switch off after usage = "False"
- No. of refined parameters = "24"
- Chi Square = "1.01018216880976"
- Rp = "0.08414"
- Rwp = "0.11624"
- Rexp = "0.11507"
- Modification time = "6/19/2019 2:18:24 PM"
- Modification editor = "TEST1"

Profile fitting:
- Angular range [°2Th.] = "4.013 - 79.959"
- Step No. 1
- Title = "Flat Background"
- Min. Shift/ESD = "0.1"
- Switch off after usage = "False"
- Step No. 2
- Title = "More background"
- Min. Shift/ESD = "0.1"
- Switch off after usage = "False"
- No. additional parameters = "3"
- Use 1/X background too = "True"
- Step No. 3
- Title = "Peak Position"
- Min. Shift/ESD = "0.1"
- Switch off after usage = "False"
- Step No. 4
- Title = "Peak Height"
- Min. Shift/ESD = "0.1"
- Switch off after usage = "False"
- Step No. 5
- Title = "Peak FWHM"
- Min. Shift/ESD = "0.1"
- Switch off after usage = "False"
- Step No. 6

- Title = "Peak Shape"
- Min. Shift/ESD = "0.1"
- Switch off after usage = "False"
- No. of refined parameters = "24"
- Chi Square = "0.99280502284823"
- Rp = "0.08245"
- Rwp = "0.11424"
- Rexp = "0.11507"
- Modification time = "6/19/2019 2:18:31 PM"
- Modification editor = "TEST1"

Delete All K-Alpha2 Peaks:
- Modification time = "6/19/2019 2:18:35 PM"
- Modification editor = "TEST1"

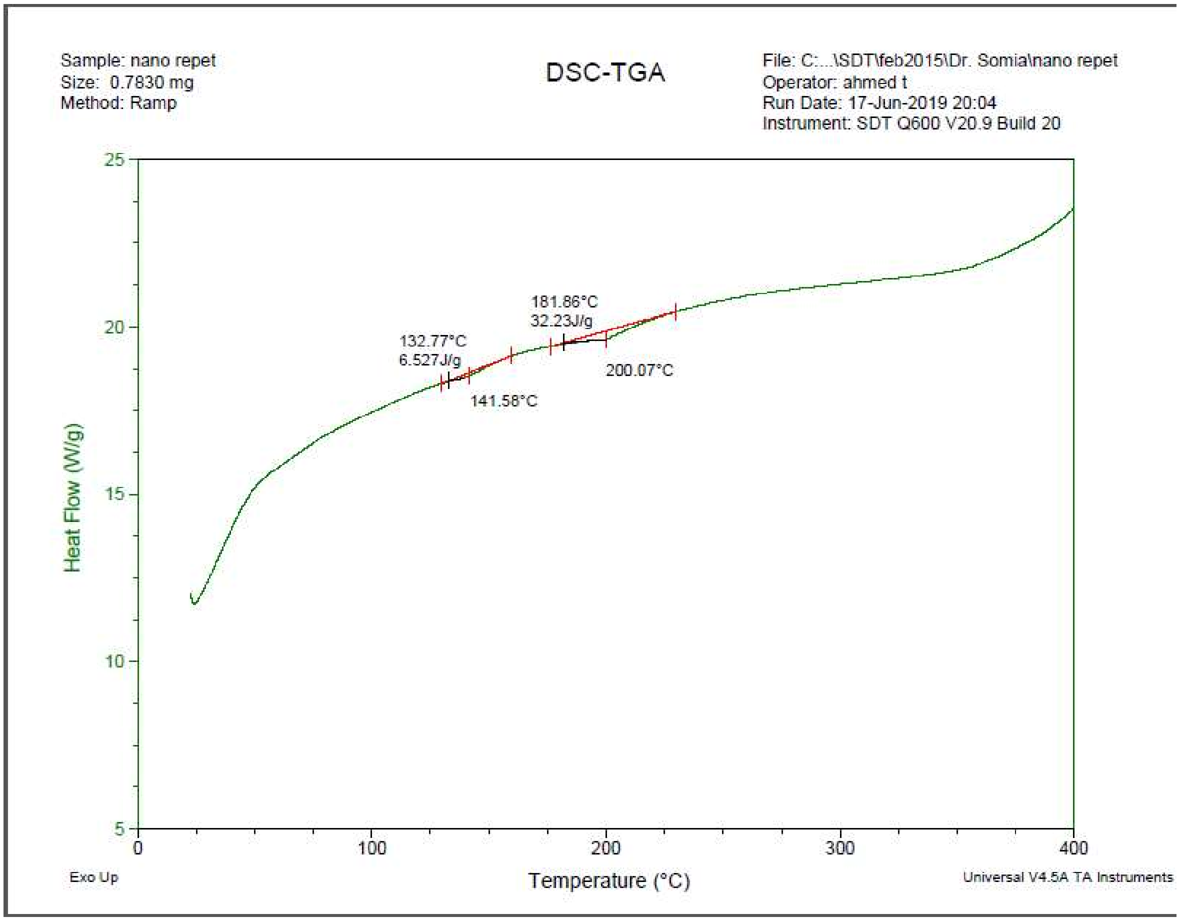

Figure S2. Analysis of **BZP-NPs**

References

1. Coskun, D.; Erkisa, M.; Ulukaya, E.; Coskun, M.F.; Ari, F. Novel 1-(7-ethoxy-1-benzofuran-2-yl) substituted chalcone derivatives: synthesis, characterization and anticancer activity. *Eur. J. Med. Chem.* **2017**, *136*, 212–222.
2. Zhang, L.; Ren, W.; Wang, X.; Zhang, J.; Liu, J.; Zhao, L.; Zhang, X. Discovery of novel polycyclic spiro-fused carbocyclicoxindole-based anticancer agents. *Eur. J. Med. Chem.* **2017**, *126*, 1071–1082.
3. Labib, M.B.; Philoppes, J.N.; Lamie, P.F.; Ahmed, E.R. Azole-hydrazone derivatives: Design, synthesis, in vitro biological evaluation, dual EGFR/HER2 inhibitory activity, cell cycle analysis and molecular docking study as anticancer agents. *Bioorg. Chem*. **2018**, *76*, 67–80.
4. Amin, K.M.; Syam, Y.M.; Anwar, M.M.; Ali, H.I.; Abdel-Ghani, TM.; Serry, A.M. Synthesis and molecular docking studies of new furochromone derivatives as p38α MAPK inhibitors targeting human breast cancer MCF-7 cells. *Bioorg. Med. Chem*. **2017**, *25*, 2423–2436.
5. Brandao, P.; Loureiro, J.B.; Carvalho, S.; Hamadou, M.H.; Cravo, S.; Moreira, J.; Pereira, D.; Palmeira, A.; Pinto, M.; Saraiva, L.; et al. Targeting the MDM2-p53 protein-protein interaction with prenylchalcones: Synthesis of a small library and evaluation of potential antitumor activity. *Eur. J. Med. Chem.* **2018**, *156*, 711–721.
6. Abdelhaleem, E.F., Abdelhameid, M.K., Kassab, A.E., Kandeel, M.M. Design and synthesis of thienopyrimidine urea derivatives with potential cytotoxic and proapoptotic activity against breast cancer cell line MCF-7. *Eur. J. Med. Chem*. **2018**, *143*, 1807–1825.
7. Jagtap P.G., Southan G.J., Baloglu E., et al. The Discovery and Synthesis of Novel Adenosine Substituted 2,3-Dihydro-1H-isoindol-1-ones: Potent Inhibitors of Poly(ADP-ribose) Polymerase-1 (PARP-1). *Bioorg. Med. Chem. Lett.* **2004**, *14*, 81–85.
